# Adverse events related to drug–drug interactions in tocilizumab combination therapy: a retrospective analysis and clinical implications

**DOI:** 10.3389/fmed.2026.1736592

**Published:** 2026-02-27

**Authors:** Ben-nian Huo, Lin Shu, Yu Zhang, Nan-ge Yin, Huan-Huan Ji, Yun-tao Jia, Lin Song

**Affiliations:** 1 Department of Pharmacy, Children’s Hospital of Chongqing Medical University, National Clinical Research Center for Children and Adolescents’ Health and Diseases, Ministry of Education Key Laboratory of Child Development and Disorders, Chongqing Key Laboratory of Child Rare Diseases in Infection and Immunity, Chongqing, China; 2 Department of Pharmacy, People’s Hospital of Chongqing Liangjiang New Area, Chongqing, China; 3 Department of Immunology, Children’s Hospital of Chongqing Medical University, Chongqing, China

**Keywords:** Adverse events, DMARDs, Drug–drug interactions, Glucocorticoids, NSAIDs, Tocilizumab

## Abstract

**Objective:**

Tocilizumab (TCZ) is widely used in the treatment of autoimmune diseases and usually needs to be combined with other drugs. However, limited evidence is available on TCZ drug–drug interactions (DDIs). This study aimed to identify and evaluate the adverse events (AEs) associated with DDIs between TCZ and co-administered drugs based on real-world clinical data to offer a reference basis for clinical decision-making.

**Methods:**

A retrospective study was conducted to estimate the AEs related to DDIs between TCZ and three categories of drugs, including disease-modifying antirheumatic drugs (DMARDs), glucocorticoids, and non-steroidal anti-inflammatory drugs (NSAIDs). AE information for the target drugs from the first quarter of 2004 to the third quarter of 2025 was downloaded from the OpenVigil FDA data platform. The following four frequency statistical models were used to detect the AEs related to DDIs and to evaluate the correlation: the reporting ratio method, the Ω shrinkage measure model, the combination risk ratio model, and the chi-squared statistics model. The influence of sex and age on the drug and the target AEs was analyzed using Pearson’s chi-squared test and reporting odds ratios with 95% confidence intervals.

**Results:**

Eleven drugs were included for the analysis of AEs associated with TCZ combination therapy. A total of 824 AEs were detected by at least one of the four models; 35.6% (293/824) AEs were significantly positively correlated with DDIs and were related to higher reporting rates of AEs than when used alone. Musculoskeletal and connective tissue disorders were the most frequently involved system organ class for TCZ-DMARD and TCZ-NSAID combinations. The most significant DDI-related AEs identified for specific TCZ drug combinations were as follows: with DMARDs (e.g., methotrexate, hydroxychloroquine, and leflunomide), occurrence of anti-cyclic citrullinated peptide (CCP) positivity, hand deformity, and pemphigus were observed; with glucocorticoids (specifically prednisone), pemphigus, hand deformity, glossodynia, and pericarditis should be monitored; with NSAIDs (specifically diclofenac), heightened vigilance is warranted for anti-CCP positive, rheumatic fever, duodenal ulcer perforation, and *Helicobacter* infections. Age and sex influence DDI risks: adults (≥18 years) were more susceptible to infusion reactions with TCZ + hydroxychloroquine or TCZ + sulfasalazine, and to joint swelling with TCZ + naproxen. Male patients demonstrated a higher incidence of stomatitis with TCZ + methotrexate, and of joint swelling with TCZ + ibuprofen or TCZ + naproxen. Female patients were more susceptible to infusion-related reactions and infections with TCZ + dexamethasone, and to abdominal discomfort with TCZ + methylprednisolone.

**Conclusion:**

Healthcare providers should maintain vigilance of potential DDI-related AEs when TCZ is used in combination with DMARDs, glucocorticoids, or NSAIDs. Particular attention is suggested for signals of decreased treatment efficacy, which may be associated with the formation of anti-TCZ antibodies, and for musculoskeletal, cutaneous, and gastrointestinal events. Monitoring serological parameters (e.g., CRP and Anti-CCP), skin/mucosal symptoms, and signs of infection is recommended during combination therapy to support medication safety.

## 1 Introduction

Tocilizumab (TCZ) is the first drug with the ability to suppress interleukin-6 receptor (IL-6R)-dependent inflammatory reactions. It was first approved in 2005 by the Pharmaceutical and Medical Devices Agency (PMDA) for moderate to severe rheumatoid arthritis (RA), followed by approvals from the European Medicines Agency (EMA) in 2009 and the U.S. Food and Drug Administration (FDA) in 2010 for patients with an inadequate response to conventional disease-modifying antirheumatic drugs (DMARDs) (1, 2). Beyond RA, TCZ has since been investigated and approved for several other immune-mediated conditions, including cytokine release syndrome, juvenile idiopathic arthritis (JIA), systemic JIA (sJIA), and giant cell arteritis (GCA) (3).

In real-world clinical practice, patients with conditions such as RA or sJIA frequently present with complex complications and usually require the prolonged use of multiple medications in combination, rather than depending exclusively on TCZ as a single-agent therapy, due to the challenges of effectively managing the condition (4–6). TCZ is frequently used in combination with non-steroidal anti-inflammatory drugs (NSAIDs), glucocorticoids, and conventional or synthetic disease-modifying antirheumatic drugs (DMARDs) (1, 7). Although this combination strategy is essential for achieving disease control, it also raises important clinical concerns regarding potential drug–drug interactions (DDIs) that may reduce the therapeutic effect of the treatment or lead to unexpected adverse outcomes. Therefore, a comprehensive understanding of potential DDIs is necessary to optimize therapeutic safety in patients receiving TCZ-based regimens.

Several studies have reported these DDI risks. For example, a large-scale, multicenter clinical trial comparing TCZ monotherapy with TCZ combined with methotrexate exhibited the highest incidence of adverse events (AEs), with 92.6% (126/136) of patients experiencing at least one AE (8). Additionally, data from phase III and IV clinical trials further indicated that a higher incidence of elevated alanine transaminase and aspartate transaminase was observed in patients receiving TCZ in combination with methotrexate compared to methotrexate alone (9). Furthermore, the combined use of TCZ and high-dose glucocorticoids has been associated with an increased risk of serious infections in pediatric patients (3).

Despite these findings, the detailed data on safety associated with DDIs between TCZ and the drugs usually used clinically in combination are still limited in the real world, owing to its relatively short time on the market and inadequate accumulation of reliable clinical data. Thus, this study aimed to utilize the Food and Drug Administration (FDA) Adverse Event Reporting System (FAERS) database to investigate DDIs between TCZ and its frequently co-prescribed medications, including DMARDs, glucocorticoids, and NSAIDs, and provide evidence supporting the safety of TCZ for clinical decision-making. Notably, the FAERS database serves as a critical resource in this study, as it provides a large volume of real-world practical data derived from post-marketing AE reports, addressing the scarcity of real-world evidence in existing TCZ DDI research (10).

## 2 Methods

### 2.1 Study design

A retrospective study was conducted to systematically evaluate AEs associated with drug interactions between TCZ and commonly co-administered medications, with a focus on identifying potential DDIs. Three drug classes were prioritized for inclusion in this study: disease-modifying antirheumatic drugs (DMARDs), glucocorticoids, and non-steroidal anti-inflammatory drugs (NSAIDs). These classes were selected because of their significant clinical safety implications and frequent use in combination with TCZ.

### 2.2 Data sources and selection criteria

Relevant data for this study were sourced from the OpenVigil FDA data platform.^1^ OpenVigil 2.1 is an open, online analysis platform built on FAERS and was used for data extraction, cleaning, mining, and analysis of pharmacovigilance data using cleansed FDA AE reporting data to detect DDIs. AEs recorded in the OpenVigil FDA database were coded using preferred terms (PTs) from the Medical Dictionary for Regulatory Activities Terminology (MedDRA version 24.1) (11). We standardized the meaningful AE reports according to the system organ classes (SOCs) of MedDRA. Only AEs with more than three reported cases (11) were further extracted for analysis of interaction-related effects between TCZ and target drugs.

### 2.3 Statistical analysis

Data from AE reports obtained via the OpenVigil FDA platform were summarized using descriptive statistical methods, with qualitative variables presented as counts and percentages. Statistical significance was set at a *p*-value of 0.05, and all analyses were conducted utilizing SPSS Statistics for Windows (version 25.0). Correlation assessments and analyses of factors influencing drug–drug interactions were performed using R software version 4.2.2 for data processing and visualization.

#### 2.3.1 Statistical models and criteria for the detection of adverse events

In this study, four frequency-based statistical models for signal mining were employed to calculate detection thresholds and identify potential AEs related to DDIs, with the criteria for AE detection set in accordance with previously published studies (12). The four frequency statistical models included the reporting ratio method, the Ω shrinkage measure model, the combination risk ratio model, and the chi-squared statistics model. The related parameters and algorithms of the four frequency statistical models are presented in Additional file 1. Cohen’s kappa coefficient was used to assess pairwise agreement in signal detection among the four frequency-based signal detection models (13). The interpretation of Cohen’s kappa is shown in Additional file 2.

#### 2.3.2 Correlation analysis

The calculation method for the relative reporting ratio (RRR) and RRR_diff values was used to quantitatively measure the correlation between drugs and target AEs with positive DDI signals, which was consistent with a previous study. Finally, the RRR_diff values (calculated from the four frequency statistical models) were used to assess the degree of correlation difference. When RRR_diff was >0.75, the correlation between the target AE and the combined use of two drugs is greater than that of either drug used alone, which indicates a significant positive correlation and leads to an increased risk of target AEs when the drugs are used in combination (14). When RRR_diff was <−0.75, the correlation between the target AE and either of the two drugs used alone is greater than when two drugs are used together, which indicates a significant negative correlation, and the risk of target AEs is greater when two drugs are used alone.

#### 2.3.3 Influencing factors analysis

The DDIs related to AEs with positive DDI signals detected by at least two of the above four models and an RRR_diff value of >0.75 were included to evaluate the influence of sex and age. Reporting odds ratios (RORs) with 95% confidence intervals (CIs) were calculated using two-by-two contingency tables. The criteria for inclusion in the statistical analysis were *a* > 5, *c* > 5, *a* + *c* > 50, with the algorithms defined as follows (see Table 1).

**Table 1:** Two-by-two contingency table used for analysis of influencing factors.

Gender/Age	Target AEs	All other AEs	Total
Male/≤18 years patients	*a*	*b*	*a + b*
Female/>18 years patients	*c*	*d*	*c* + *d*
Total	*a* + *c*	*b* + *d*	*n* (*a* + *b* + *c* + *d*)

AEs, adverse events.

*a*: the number of male (or ≤18 years) patients with target adverse events. *b*: the number of male (or ≤18 years) patients without target events. *c*: the number of female patients (or >18 years) with target adverse events. *d*: the number of female patients without target adverse events. Reporting odds ratio (ROR) = (*ad*)/(*bc*) $95\;\mathrm{CI}=e^{\ln(\mathrm{ROR})\pm 1.96\sqrt{1/a+1/b+1/c+1/d}}$ .

Pearson’s chi-squared test was used to compare the differences in the risk of target AEs between female and male patients and between patients aged ≤18 years and those >18 years. The standards for statistical significance were as follows: a Pearson’s chi-squared test with a *p*-value of <0.05 (−log_10_ *p*-value >1.301) and a log_2_ROR of >1 indicates a higher risk of target AEs in male patients or patients aged ≤18 years; a *p*-value of <0.05 (−log_10_ *p*-value >1.301) and a log_2_ROR of <−1 indicates a higher risk of target AEs in female patients or patients aged >18 years (15).

## 3 Results

### 3.1 General results

This study included three categories of drugs commonly used in combination with TCZ, which were included in this study for the detection of AEs related to DDIs. A total of 11 drugs were included; the details were as follows:

1. DMARDs: methotrexate, leflunomide, hydroxychloroquine, and sulfasalazine.
2. Glucocorticoids: prednisone, methylprednisolone, and dexamethasone.
3. NSAIDs: celecoxib, diclofenac, ibuprofen, and naproxen.

**Figure 1:**
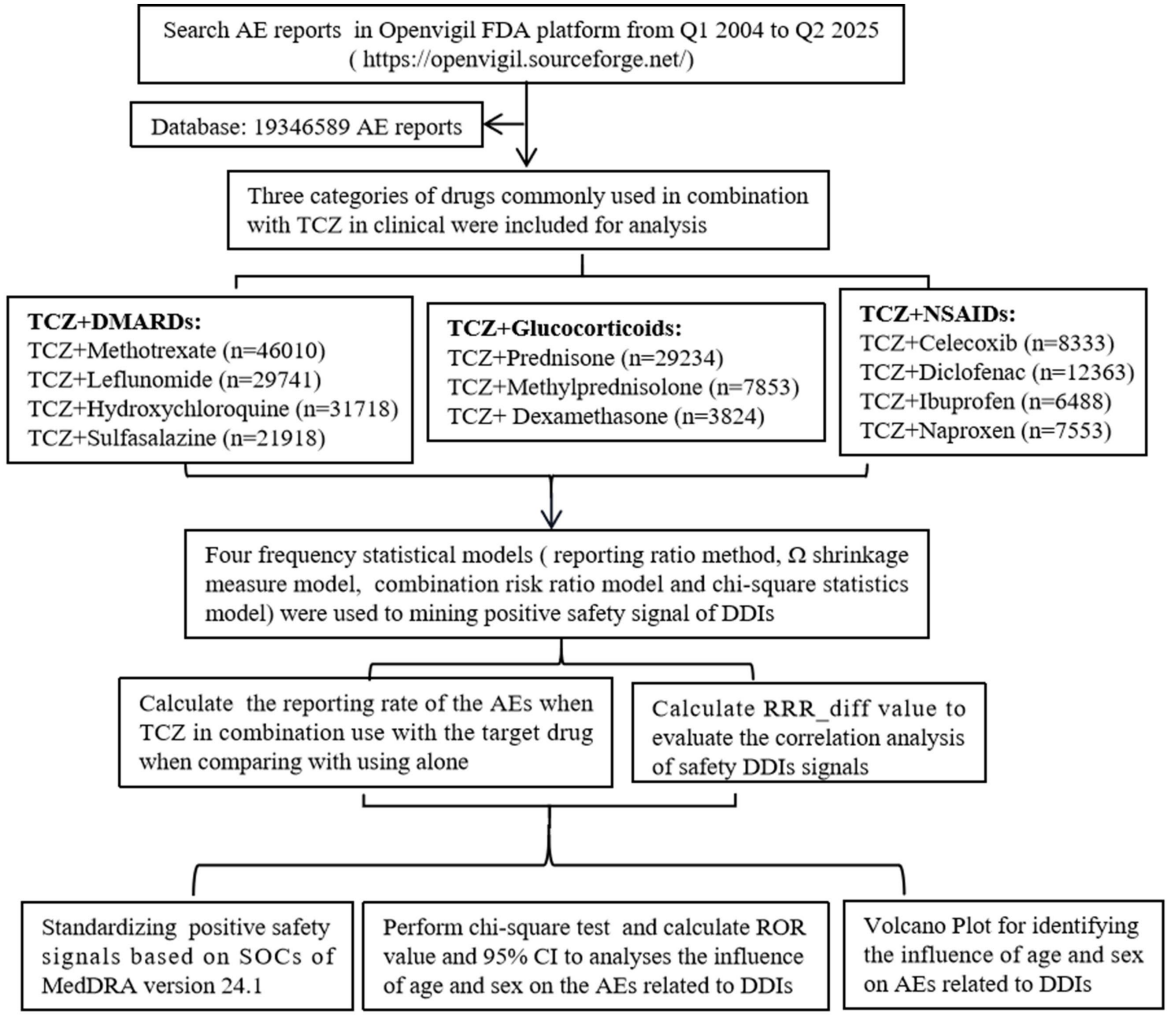
Flowchart of the inclusion of adverse events. AE, adverse event; DDIs, drug–drug interactions; DMARDs, disease-modifying antirheumatic drugs; NSAIDs, non-steroidal anti-inflammatory drugs; TCZ, tocilizumab; RRR, relative reporting ratio; ROR, reporting odds ratio; MedDRA, Medical Dictionary for Regulatory Activities Terminology; SOC, system organ classes.

A total of 19,346,589 AE reports were recorded in the FAERS database on the OpenVigil FDA platform as of 30 September 2025. Among these, 129,387, 40,911, and 34,737 AE reports were recorded when TCZ was used in combination with DMARDs, glucocorticoids, and NSAIDs, respectively. The flowchart is shown in Figure 1.

### 3.2 TCZ adverse event signals related to DDIs

**Figure 2:**
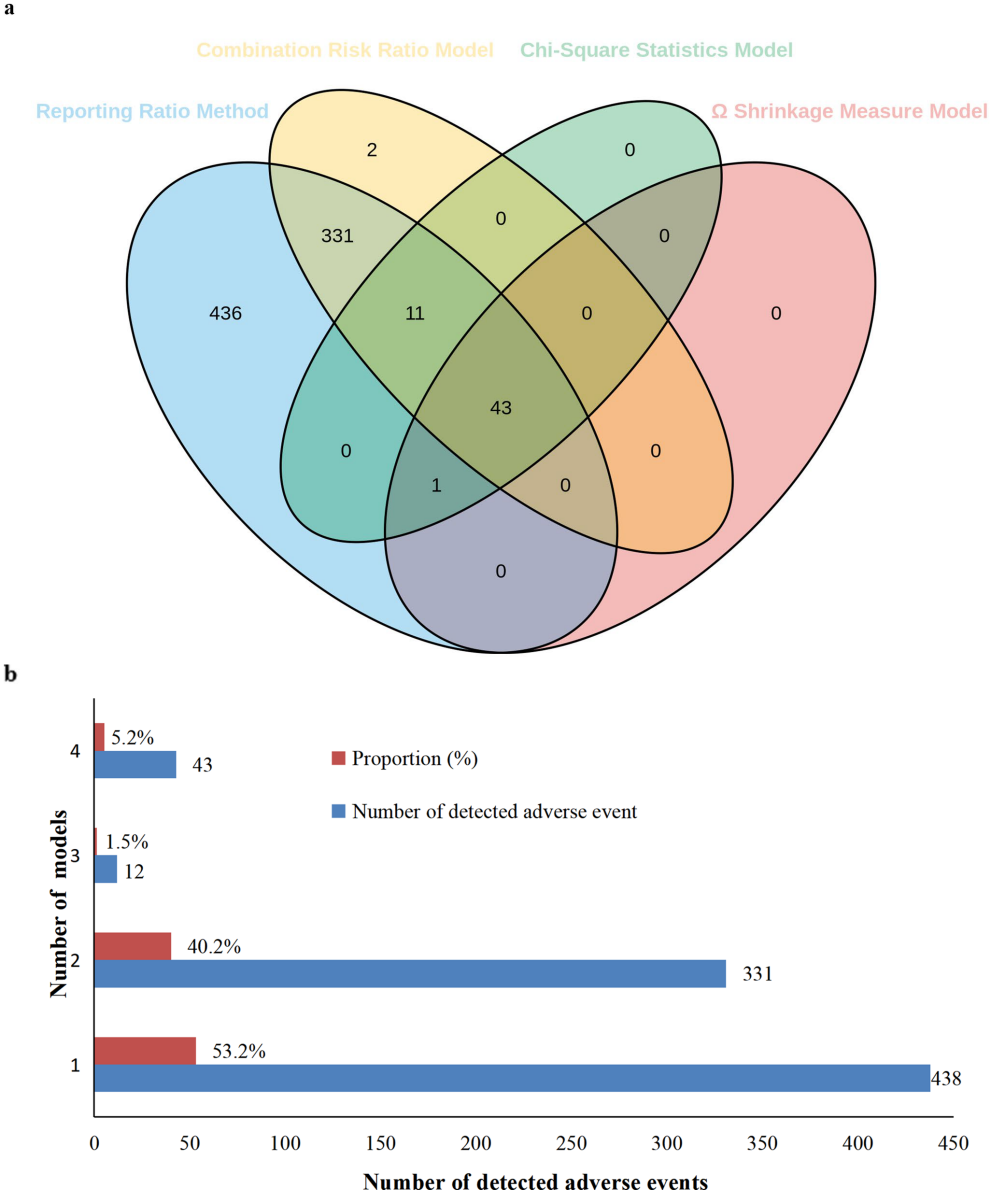
Inter-model agreement and signal detection overlap in adverse event mining. **(a)** Venn diagram of adverse event signals detected by four frequency statistical models. The diagram illustrates the number of unique and overlapping signals generated by four frequency statistical models: the reporting ratio method (blue), the Ω shrinkage measure model (pink), the combination risk ratio model (yellow), and the chi-squared statistics model (green). The numbers in the diagram represent the count of adverse events uniquely detected by each method or shared among them, with 43 adverse events detected by all four models together. **(b)** The distribution of adverse events detected by different numbers of models. This bar chart presents the proportion and absolute count of adverse events identified by 1, 2, 3, or 4 models. Red bars represent the percentage of total signals, and blue bars indicate the corresponding number of events. The predominance of signals detected by only one model underscores the importance of multi-model consensus strategies to improve signal reliability, while the subset detected by multiple models suggests higher-confidence associations worthy of closer clinical scrutiny.

The number of AE categories, positive safety signals, and the age distribution of the AEs when TCZ was used in combination with the target drugs are presented in Table 2. A total of 824 AEs were detected by at least one of the four frequency statistical models when TCZ and 11 drugs were co-administered, and the results are provided in Additional file 3. The number of unique and overlapping AE signals generated by the four frequency statistical models is illustrated in the Venn diagram shown in Figure 2a, and the distribution of AEs detected by different numbers of models is shown in Figure 2b, with 43 AE signals detected by all four frequency statistical models. The consistency analysis results of the four frequency statistical models are detailed in Table 3. The results revealed notable variations in consistency across the different model pairs. Specifically, the Ω shrinkage measure model and the chi-squared Statistics Model demonstrated very strong agreement (Kappa = 0.8819), indicating a high level of synergy between the two models in identifying potential drug–drug interaction signals. In contrast, other model combinations, such as the reporting ratio method and the Ω shrinkage measure model, showed weaker agreement or levels close to chance.

**Table 2:** Number of AE categories, positive safety signals, and the gender and age distribution of the AEs when TCZ is used in combination with the target drugs^a^.

Drug combination	Count both drugs^b^	AE categories	Positive DDI signals^c^	Male	Female	≤18 years	>18 years
TCZ + DMARDs	**129,387**	**370**	**355**	**96,918**	**14,176**	**2,527**	**68,479**
Methotrexate	46,010	82	77	34,212	5,960	1,425	24,052
Leflunomide	29,741	97	93	22,768	2,674	287	15,679
Hydroxychloroquine	31,718	95	91	23,350	3,761	475	17,251
Sulfasalazine	21,918	96	94	16,588	1,781	340	11,497
TCZ + Glucocorticoids	**40,911**	**182**	**161**	**28,576**	**6,355**	**1,295**	**23,874**
Prednisone	29,234	75	73	21,973	3,113	656	16,053
Methylprednisolone	7,853	62	60	4,870	1,587	431	4,869
Dexamethasone	3,824	45	28	1,733	1,655	208	2,952
TCZ + NSAIDs	**34,737**	**314**	**308**	**27,436**	**1,487**	**462**	**20,276**
Celecoxib	8,333	79	78	6,504	437	67	5,141
Diclofenac	12,363	89	87	9,774	395	54	6,395
Ibuprofen	6,488	66	64	5,188	294	90	3,978
Naproxen	7,553	80	79	5,970	361	251	4,762
Total	205,035	866	824	152,930	22,018	4,284	112,629

Data was presented as number.

AE, adverse event; DDIs, drug–drug interactions; DMARDs, disease-modifying antirheumatic drugs; NSAIDs, non-steroidal anti-inflammatory drugs; TCZ, tocilizumab.

a Due to the technical limitations and the constant changes of the OpenVigil FDA API, the unbalanced of the data extracted from the database was about 16% in this study.

b The reported number of target AEs when two drugs are used together.

c Positive DDI signals detected by at least one of the four frequency statistical models when TCZ used in combination with target drug.

Bold text indicates the summary data for the main drug combination categories (TCZ+DMARDs, TCZ+Glucocorticoids, TCZ+NSAIDs) under each data column, helping to differentiate the hierarchical structure of the table (such as main categories and summaries) and highlight the most important data categories.

**Table 3:** Model pairwise agreement and overlap results.

Model A	Model B	Cohen’s kappa (*κ*)^a^	Number of signals co-detected by model A and model B	Pairwise overlap (%)^b^
Reporting ratio method	Ω shrinkage measure model	0.0003	44	5.34
Reporting ratio method	Combination risk ratio model	−0.0049	385	46.72
Reporting ratio method	Chi-square statistics model	0.0003	55	6.67
Ω shrinkage measure model	Combination risk ratio model	0.1146	43	5.22
Ω shrinkage measure model	Chi-square statistics model	0.8819	44	5.34
Combination risk ratio model	Chi-square statistics model	0.1443	54	6.55

a Cohen’s kappa is suitable for assessing pairwise inter-model agreementt, the calculation formula for Cohen’s kappa is detailed in reference (13), the interpretation of Cohen’s kappa was shown in Additional file 2.

b $\text{Pairwise overlap}\%=\frac{\;\text{Number of}\;\mathrm{co}-\text{detected signals}}{\text{Total signals in Model}\;A\;\text{or}\;B}$ × 100%.

The most common DDI-related AEs for TCZ combined with DMARDs, glucocorticoids, and NSAIDs were increased C-reactive protein [2.0% (7/355)] and abdominal discomfort [1.1% (4/355)], condition aggravated [1.9% (3/161)] and abdominal discomfort [1.9% (3/161)], and hypersensitivity [1.6% (5/308)] and infusion-related reaction [1.6% (5/308)], respectively.

### 3.3 Correlation analysis

After calculating the RRR_diff values of 824 AE signals, a total of 293 target AEs [35.6% (293/824)] showed a significantly positive correlation with DDIs during concomitant use of TCZ, indicating increased AE risks. These 293 target AEs were further classified by SOCs; the specific details are listed in Table 4. The results indicated that the positive signals generated primarily involved 14 SOCs: musculoskeletal and connective tissue disorders accounted for the highest proportion (8.9%, 26/293) when TCZ was combined with DMARDs and NSAIDs (10.9%, 32/293), respectively, whereas general disorders and administration site conditions represented the highest proportion in TCZ combinations with glucocorticoids (6.8%, 20/293).

**Table 4:** A total of 293 adverse reaction signals had a positive correlation with DDIs when TCZ was used in combination with the target drugs, categorized by system organ class.

System organ class	Total	TCZ + DMARDs	TCZ + GS	TCZ + NSAIDs
Musculoskeletal and connective tissue disorders	70 (23.9)	26 (8.9)	12 (4.1)	32 (10.9)
General disorders and administration site conditions	66 (22.5)	19 (6.5)	20 (6.8)	27 (9.2)
Skin and subcutaneous tissue disorders	30 (10.2)	16 (5.5)	3 (1.0)	11 (3.8)
General investigations	28 (9.6)	15 (5.1)	3 (1.0)	10 (3.4)
Immune system disorders	26 (8.9)	10 (3.4)	8 (2.7)	8 (2.7)
Gastrointestinal disorders	24 (8.2)	10 (3.4)	7 (2.4)	7 (2.4)
Nervous system disorders	18 (6.1)	6 (2.0)	5 (1.7)	7 (2.4)
Cardiac disorders	10 (3.4)	4 (1.4)	3 (1.0)	3 (1.0)
Respiratory, thoracic and mediastinal disorders	7 (2.4)	2 (0.7)	2 (0.7)	3 (1.0)
Hepatobiliary disorders	4 (1.4)	1 (0.3)	/	3 (1.0)
Metabolic and nutritional disorders	4 (1.4)	1 (0.3)	/	3 (1.0)
Pregnancy, puerperium and perinatal conditions	2 (0.7)	/	2 (0.7)	/
Infections and infestations	2 (0.7)	/	1 (0.3)	1 (0.3)
Product issues	2 (0.7)	/	2 (0.7)	/

Data are presented as number (percentage).

DDIs, drug–drug interactions; DMARDs, disease-modifying antirheumatic drugs; NSAIDs, non-steroidal anti-inflammatory drugs; TCZ, tocilizumab; GS, glucocorticoids.

**Figure 3:**
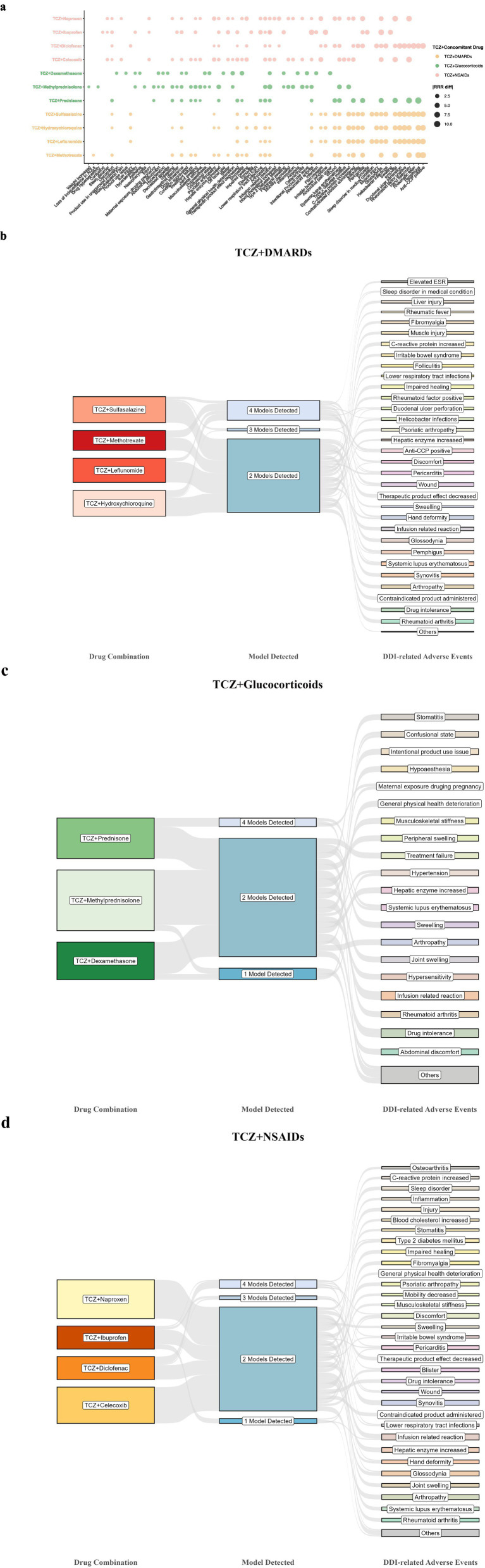
Correlation analysis results between the drug(s) and the target AEs with positive DDI signals. **(a)** A bubble chart depicting the correlation analysis results of the 293 adverse events. **(b–d)** The four frequency statistical model distributions of 293 adverse events positively correlated with DDIs when TCZ was combined with target drugs. The larger the bubble is, the greater the absolute value of the RRR_diff value, indicating a greater correlation between the drug(s) and the target AE. ADL, activities of daily living; Anti-CCP, anti-cyclic citrullinated peptide; DDIs, drug–drug interactions; DMARDs, disease-modifying antirheumatic drugs; ESR, erythrocyte sedimentation rate; NSAIDs, non-steroidal anti-inflammatory drugs; TCZ, tocilizumab; RRR, relative reporting ratio.

The correlation analysis results between the target drug and the target AEs with positive DDI signals are shown in Figure 3a. The four frequency statistical model distributions of these 293 AEs are presented in Figures 3b–d, and based on the RRR_diff value, the results of the top 20 adverse event signals with positive DDI correlation for each of the three TCZ combinations are detailed in Table 5. For a specific positive signal result, reliability increased with the number of detecting models, and a higher RRR_diff value indicated a stronger correlation between the drug and the target AE. The results revealed that, when TCZ was combined with DMARDs (such as methotrexate, hydroxychloroquine, or leflunomide), all four models indicated anti-CCP positive and pemphigus as the AEs that had the strongest correlation with the drug interaction. Furthermore, special attention should be paid to hand deformity when TCZ is used in combination with methotrexate or hydroxychloroquine. When TCZ was combined with glucocorticoids, all four models revealed specific AEs related to DDIs. Prednisone showed the strongest associations with pemphigus, hand deformity, glossodynia, pericarditis, systemic lupus erythematosus, and wounds. When TCZ was combined with NSAIDs, particularly diclofenac, all four models showed that special attention should be paid to the presence of anti-CCP positive, rheumatic fever, duodenal ulcer perforation, and *Helicobacter* infections. For ibuprofen, it was recommended to carefully monitor for irritable bowel syndrome. Notably, compared with TCZ monotherapy, the reporting rates of all 293 AEs with significant positively correlation with DDIs increased when TCZ was co-administered with the target drugs, indicating that close attention should be paid to these target AEs.

**Table 5:** Top 20 adverse event signals with positive DDI correlation for each of the three TCZ combinations, ranked by RRR_diff value^a^.

Drug combination	Adverse event (PT)	Count both drugs^b^	RRR_diff^c^	Reporting rate (%)			Four frequency statistical models^d^			
				TCZ	Target drug	Both drugs	Reporting ratio method	Ω shrinkage measure model	Combination risk ratio model	Chi-square statistics model
TCZ + DMARDs										
TCZ + Methotrexate	Anti-CCP positive	5,039	9.2	0.6	0.1	11.0	Yes	Yes	Yes	Yes
TCZ + Methotrexate	Pemphigus	8,076	8.4	0.6	0.1	17.6	Yes	Yes	Yes	Yes
TCZ + Hydroxychloroquine	Anti-CCP positive	4,941	7.1	0.6	0.3	15.6	Yes	Yes	Yes	Yes
TCZ + Sulfasalazine	Anti-CCP positive	4,789	6.9	0.7	0.5	21.8	Yes	No	Yes	Yes
TCZ + Leflunomide	Anti-CCP positive	5,057	6.8	0.4	0.5	17.0	Yes	Yes	Yes	Yes
TCZ + Hydroxychloroquine	Pemphigus	7,987	6.5	0.6	0.3	25.2	Yes	Yes	Yes	Yes
TCZ + Methotrexate	Hand deformity	6,844	6.4	0.8	0.3	14.9	Yes	Yes	Yes	Yes
TCZ + Hydroxychloroquine	Rheumatoid factor positive	3,780	6.3	0.7	0.3	11.9	Yes	No	Yes	No
TCZ + Sulfasalazine	Rheumatic fever	2,492	6.2	0.4	0.2	11.4	Yes	No	Yes	No
TCZ + Leflunomide	Pemphigus	8,061	6.2	0.5	0.5	27.1	Yes	Yes	Yes	Yes
TCZ + Methotrexate	Synovitis	9,182	6.1	1.1	0.8	20.0	Yes	No	Yes	No
TCZ + Sulfasalazine	Rheumatoid factor positive	3,585	6.0	0.8	0.4	16.4	Yes	No	Yes	No
TCZ + Leflunomide	Rheumatoid factor positive	3,758	5.9	0.7	0.5	12.6	Yes	No	Yes	No
TCZ + Sulfasalazine	Pemphigus	7,123	5.7	1.5	0.5	32.5	Yes	No	Yes	No
TCZ + Leflunomide	Rheumatic fever	2,466	5.7	0.4	0.3	8.3	Yes	No	Yes	No
TCZ + Sulfasalazine	Duodenal ulcer perforation	3,892	5.6	0.4	0.4	17.8	Yes	Yes	Yes	Yes
TCZ + Hydroxychloroquine	Duodenal ulcer perforation	3,866	5.5	0.5	0.3	12.2	Yes	No	Yes	Yes
TCZ + Leflunomide	Duodenal ulcer perforation	3,918	5.2	0.4	0.6	13.2	Yes	No	Yes	No
TCZ + Hydroxychloroquine	Hand deformity	6,772	5.1	0.7	0.5	21.4	Yes	Yes	Yes	Yes
TCZ + Sulfasalazine	Hand deformity	6,434	4.8	1.0	0.9	29.4	Yes	No	Yes	No
TCZ + Glucocorticoids										
TCZ + Prednisone	Pemphigus	7,760	10.1	0.9	0.1	26.5	Yes	Yes	Yes	Yes
TCZ + Prednisone	Hand deformity	6,525	7.8	1.0	0.2	22.3	Yes	Yes	Yes	Yes
TCZ + Prednisone	Synovitis	7,087	5.7	3.6	0.2	24.2	Yes	No	Yes	No
TCZ + Prednisone	Glossodynia	7,292	5.1	0.7	0.1	24.9	Yes	Yes	Yes	Yes
TCZ + Prednisone	Pericarditis	5,077	4.9	0.5	0.2	17.4	Yes	Yes	Yes	Yes
TCZ + Methylprednisolone	Infusion related reaction	2,149	4.4	6.6	3.0	27.4	Yes	No	Yes	No
TCZ + Methylprednisolone	Systemic lupus erythematosus	1,148	3.9	7.9	0.5	14.6	Yes	No	Yes	No
TCZ + Prednisone	Contraindicated product administered	8,048	3.9	4.4	0.3	27.5	Yes	No	Yes	No
TCZ + Methylprednisolone	C-reactive protein increased	1,030	3.9	2.9	1.0	13.1	Yes	No	Yes	No
TCZ + Prednisone	Systemic lupus erythematosus	7,985	3.6	1.2	0.6	27.3	Yes	Yes	Yes	Yes
TCZ + Methylprednisolone	Hepatic enzyme increased	1,693	3.4	6.4	0.6	21.6	Yes	No	Yes	No
TCZ + Prednisone	Wound	5,971	3.3	0.7	0.3	20.4	Yes	Yes	Yes	Yes
TCZ + Dexamethasone	Stomatitis	250	3.3	4.6	0.7	6.5	Yes	No	Yes	No
TCZ + Methylprednisolone	Osteoarthritis	865	2.8	2.3	0.7	11.0	Yes	No	Yes	No
TCZ + Dexamethasone	General physical health deterioration	327	2.8	4.0	1.0	8.6	Yes	No	Yes	No
TCZ + Methylprednisolone	Inflammation	901	2.4	2.7	0.6	11.5	Yes	No	Yes	No
TCZ + Dexamethasone	Intentional product use issue	256	2.4	4.5	0.8	6.7	Yes	No	Yes	No
TCZ + Dexamethasone	Abdominal pain upper	436	2.4	3.5	0.7	11.4	Yes	No	Yes	No
TCZ + Methylprednisolone	Arthropathy	1,206	2.3	9.1	0.6	15.4	Yes	No	Yes	No
TCZ + Methylprednisolone	Rheumatoid arthritis	2,483	2.3	19.4	1.8	31.6	Yes	No	Yes	No
TCZ + NSAIDs										
TCZ + Diclofenac	Anti-CCP positive	4,645	7.5	0.8	0.3	37.6	Yes	Yes	Yes	Yes
TCZ + Celecoxib	Hand deformity	1,743	7.2	5.7	0.2	20.9	Yes	No	Yes	No
TCZ + Diclofenac	Rheumatic fever	2,531	7.1	0.3	0.2	20.5	Yes	Yes	Yes	Yes
TCZ + Naproxen	Synovitis	2,982	7.1	6.9	0.4	39.5	Yes	No	Yes	No
TCZ + Ibuprofen	Glossodynia	2,471	7.0	3.0	0.1	38.1	Yes	No	Yes	No
TCZ + Naproxen	Hand deformity	2,084	6.9	5.3	0.2	27.6	Yes	No	Yes	No
TCZ + Diclofenac	Duodenal ulcer perforation	3,949	6.4	0.3	0.4	31.9	Yes	Yes	Yes	Yes
TCZ + Ibuprofen	Rheumatoid arthritis	2,548	6.0	7.3	0.2	39.3	Yes	No	Yes	No
TCZ + Diclofenac	Pemphigus	6,606	6.0	1.9	0.4	53.4	Yes	No	Yes	No
TCZ + Celecoxib	Synovitis	2,060	5.9	7.9	0.4	24.7	Yes	No	Yes	No
TCZ + Diclofenac	Rheumatoid factor positive	3,053	5.7	1.3	0.2	24.7	Yes	No	Yes	No
TCZ + Ibuprofen	Irritable bowel syndrome	2,526	5.5	1.5	0.2	38.9	Yes	Yes	Yes	Yes
TCZ + Diclofenac	Hand deformity	5,928	5.0	1.5	0.4	47.9	Yes	No	Yes	Yes
TCZ + Naproxen	Pericarditis	1,801	4.9	3.7	0.4	23.8	Yes	No	Yes	No
TCZ + Diclofenac	*Helicobacter* infections	3,994	4.5	0.4	0.3	32.3	Yes	Yes	Yes	Yes
TCZ + Ibuprofen	Swelling	2,302	4.5	5.5	0.2	35.5	Yes	No	Yes	No
TCZ + Celecoxib	Fibromyalgia	1,798	4.3	1.7	0.9	21.6	Yes	No	Yes	No
TCZ + Celecoxib	Glossodynia	1,707	4.0	6.3	0.2	20.5	Yes	No	Yes	No
TCZ + Naproxen	Glossodynia	2,051	3.8	5.9	0.2	27.2	Yes	No	Yes	No
TCZ + Naproxen	Fibromyalgia	2,058	3.8	1.5	0.6	27.2	Yes	No	Yes	No

Anti-CCP, anti-cyclic citrullinated peptide antibody; DDIs, drug–drug interactions; PT, preferred term; DMARDs, disease-modifying antirheumatic drugs; NSAIDs, non-steroidal anti-inflammatory drugs; TCZ, tocilizumab.

a A total of 293 target AEs showed a significantly positive correlation with DDIs during concomitant use of TCZ, only the top 20 AEs ranked by RRR_diff value for each drug category were listed in Table 5.

b The reported number of target AEs when two drugs are used together.

c When the RRR_diff value >0.75, indicate that the target AE had positive correlation with DDIs when TCZ was used in combination. The higher the RRR_diff value was, the greater the correlation between the drug(s) and the target AE.

d Statistically significant results are denoted by “Yes,” while results without statistical significance are denoted by “No”.

### 3.4 Impact of age and sex on DDI-related adverse events

**Figure 4:**
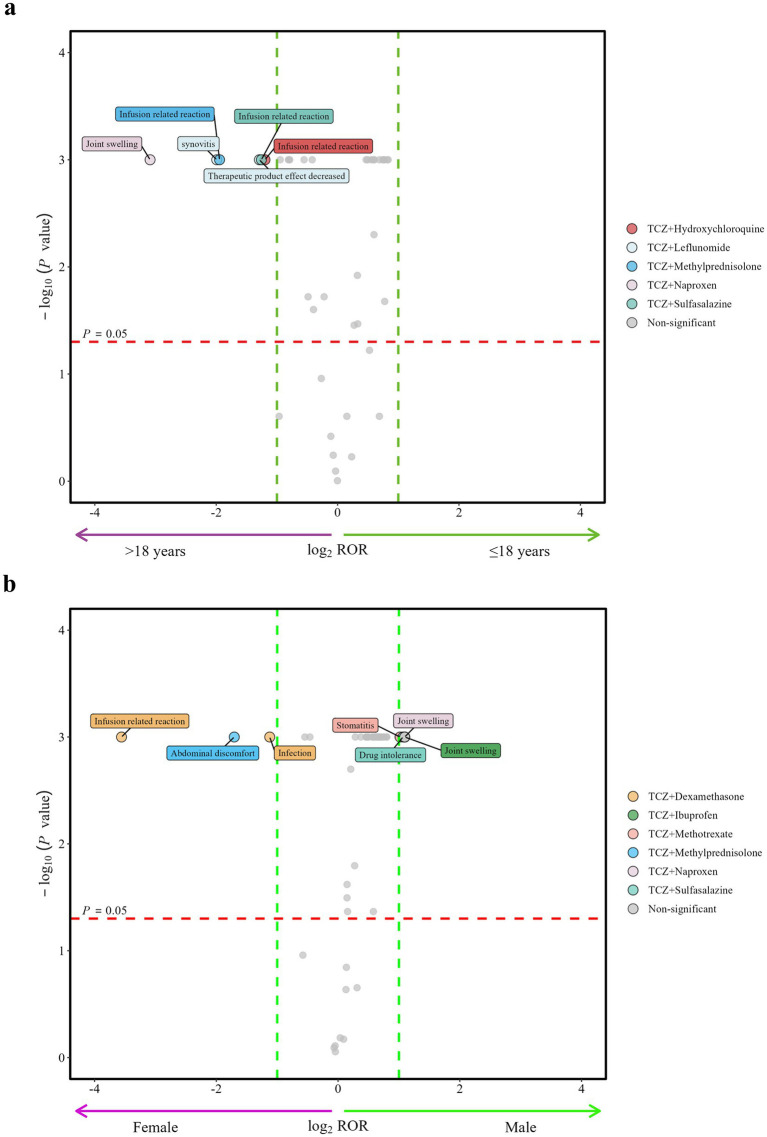
Influence of age and sex on the incidence of adverse events related to drug–drug interactions: **(a)** age and **(b)** sex. Each spot represents a specific AE related to drug–drug interactions. ROR, reporting odds ratio.

The impact of age and sex on the above 293 AEs positively correlated with DDIs was further analyzed, and only AEs that were confirmed by at least two of the four frequency statistical models were included. Ultimately, 41 target AEs in the age group and 53 in the sex group met the inclusion criteria for statistical assessment of influencing factors. Other AEs were excluded primarily due to insufficient case numbers required for statistical evaluation. Relevant results are summarized in Tables 6, 7 and Figures 4a,b.

**Table 6:** Comparison of the risk of the AEs related to DDIs between patients aged ≤18 years and those aged >18 years.

Drugs	Adverse event (PT)	≤18 years patients		>18 years patients		*χ*	*p*	ROR (95% CI)	Log_2_ROR
		Target AE	Other AEs	Target AE	Other AEs				
TCZ + DMARDs									
TCZ + Methotrexate	Rheumatoid arthritis	246	5,494	13,431	257,091	5.5	0.019	0.86 (0.75–0.98)	−0.222
TCZ + Methotrexate	Drug intolerance	208	5,532	6,584	263,938	33.2	<0.000	1.51 (1.31–1.73)	0.592
TCZ + Methotrexate	Treatment failure	140	5,600	4,716	265,806	15.8	<0.000	1.41 (1.19–1.67)	0.495
TCZ + Methotrexate	Infusion related reaction	76	5,664	4,303	266,219	2.6	0.110	0.83 (0.66–1.04)	−0.269
TCZ + Leflunomide	Drug intolerance	88	2,215	5,930	262,329	27.2	<0.000	1.76 (1.42–2.18)	0.814
TCZ + Leflunomide	Contraindicated product administered	67	2,236	4,655	263,604	18.4	<0.000	1.70 (1.33–2.17)	0.763
**TCZ + Leflunomide**	**Synovitis**	11	2,292	5,023	263,236	24.3	<**0.000**	0.25 (0.14–0.46)	**−1.991**
TCZ + Leflunomide	Infusion related reaction	47	2,256	3,640	264,619	7.9	0.005	1.51 (1.13–2.03)	0.599
**TCZ + Leflunomide**	**Therapeutic product effect decreased**	13	2,290	3,669	264,590	11.0	**0.001**	0.41 (0.24–0.71)	**−1.288**
TCZ + Hydroxychloroquine	Rheumatoid arthritis	128	6,082	7,919	256,483	18.3	<0.000	0.68 (0.57–0.81)	−0.553
TCZ + Hydroxychloroquine	Drug intolerance	134	6,076	6,153	258,249	0.8	0.381	0.93 (0.78–1.10)	−0.111
TCZ + Hydroxychloroquine	Contraindicated product administered	131	6,079	4,636	259,766	4.4	0.035	1.21 (1.01–1.44)	0.272
TCZ + Hydroxychloroquine	Arthropathy	172	6,038	4,583	259,819	37.8	<0.000	1.61 (1.38–1.88)	0.691
TCZ + Hydroxychloroquine	Synovitis	122	6,088	5,312	259,090	0.1	0.805	0.98 (0.82–1.17)	−0.033
**TCZ + Hydroxychloroquine**	**Infusion related reaction**	38	6,172	3,679	260,723	27.2	<**0.000**	0.44 (0.32–0.60)	**−1.197**
TCZ + Hydroxychloroquine	Therapeutic product effect decreased	49	6,161	3,587	260,815	14.7	<0.000	0.58 (0.44–0.77)	−0.790
TCZ + Sulfasalazine	Rheumatoid arthritis	131	6,031	6,505	224,051	10.7	0.001	0.75 (0.63–0.89)	−0.419
TCZ + Sulfasalazine	Drug intolerance	134	6,028	5,265	225,291	0.3	0.572	0.95 (0.80–1.13)	−0.072
TCZ + Sulfasalazine	Contraindicated product administered	131	6,031	3,927	226,629	6.4	0.012	1.25 (1.05–1.49)	0.326
TCZ + Sulfasalazine	Arthropathy	176	5,986	3,921	226,635	47.1	<0.000	1.70 (1.46–1.98)	0.765
TCZ + Sulfasalazine	Synovitis	126	6,036	4,251	226,305	1.3	0.248	1.11 (0.93–1.33)	0.152
**TCZ + Sulfasalazine**	**Infusion related reaction**	39	6,123	3,455	227,101	30.9	<**0.000**	0.42 (0.31–0.57)	**−1.256**
TCZ + Sulfasalazine	Therapeutic product effect decreased	49	6,113	3,167	227,389	15.0	<0.000	0.58 (0.43–0.76)	−0.797
TCZ + Glucocorticoids									
TCZ + Prednisone	Drug intolerance	141	4,220	4,339	197,811	23.8	<0.000	1.52 (1.28–1.81)	0.607
TCZ + Prednisone	Synovitis	126	4,235	4,251	197,899	12.7	<0.000	1.39 (1.16–1.66)	0.470
TCZ + Prednisone	Treatment failure	45	4,316	3,631	198,519	14.3	<0.000	0.57 (0.42–0.77)	−0.811
TCZ + Prednisone	Infusion related reaction	39	4,322	3,455	198,695	17.0	<0.000	0.52 (0.38–0.71)	−0.946
TCZ + Prednisone	Therapeutic product effect decreased	49	4,312	3,167	198,983	5.5	0.019	0.71 (0.54–0.95)	−0.486
**TCZ + Methylprednisolone**	**Infusion related reaction**	7	917	1,357	46,161	14.6	<**0.000**	0.26 (0.12–0.55)	**−1.945**
TCZ + Methylprednisolone	Hypertension	28	896	1,010	46,508	3.5	0.060	1.44 (0.98–2.11)	0.525
TCZ + Methylprednisolone	Intentional product use issue	19	905	576	46,942	5.3	0.021	1.71 (1.08–2.71)	0.775
TCZ + Dexamethasone	Condition aggravated	12	352	314	10,811	0.3	0.592	1.17 (0.65–2.11)	0.231
TCZ + Dexamethasone	Infusion related reaction	4	360	236	10,889	1.3	0.248	0.51 (0.19–1.38)	−0.964
TCZ + NSAIDs									
TCZ + Celecoxib	Rheumatoid arthritis	6	140	2,378	89,422	1.3	0.248	1.61 (0.71–3.65)	0.688
TCZ + Naproxen	Rheumatoid arthritis	69	4,001	2,240	98,603	5.0	0.025	0.76 (0.60–0.97)	−0.398
**TCZ + Naproxen**	**Joint swelling**	9	4,061	1,867	98,976	59.2	<**0.000**	0.12 (0.06–0.23)	**−3.089**
TCZ + Naproxen	Drug intolerance	108	3,962	1,520	99,323	33.6	<0.000	1.78 (1.46–2.17)	0.833
TCZ + Naproxen	Contraindicated product administered	57	4,013	1,415	99,428	0.0	0.989	1.00 (0.76–1.30)	−0.003
TCZ + Naproxen	Arthropathy	91	3,979	1,800	99,043	4.5	0.034	1.26 (1.02–1.56)	0.332
TCZ + Naproxen	Synovitis	117	3,953	1,990	98,853	16.1	<0.000	1.47 (1.22–1.78)	0.556
TCZ + Naproxen	Mobility decreased	97	3,973	1,449	99,394	24.1	<0.000	1.67 (1.36–2.06)	0.744

AE, adverse event; CI, confidence interval; DDIs: drug–drug interactions; DMARDs, disease-modifying antirheumatic drugs; PT, preferred term; ROR, reporting odds ratio; NSAIDs, non-steroidal anti-inflammatory drugs; TCZ, tocilizumab.

Statistically significant results are highlighted in bold: log_2_ROR >1 or log_2_ROR <−1, and Pearson’s chi-square test with *p* < 0.05. ROR >1 indicates the ≤18 years is more likely to have a higher risk of target AEs, and 0 < ROR < 1 indicates the >18 years patients tends to have a higher risk of target AEs.

**Table 7:** Comparison of the risk of the AEs related to DDIs between male and female patients.

Drug combination	Adverse event (PT)	Male		Female		*χ*	*p*	ROR (95% CI)	Log_2_ROR
		Target AE	Other AEs	Target AE	Other AEs				
TCZ + DMARDs									
TCZ + Methotrexate	Rheumatoid arthritis	1,329	22,726	14,214	382,236	239.7	<0.000	1.57 (1.48–1.67)	0.653
TCZ + Methotrexate	Joint swelling	882	23,173	9,646	386,804	141.4	<0.000	1.53 (1.42–1.64)	0.610
TCZ + Methotrexate	Drug intolerance	731	23,324	8,796	387,654	68.9	<0.000	1.38 (1.28–1.49)	0.466
TCZ + Methotrexate	Contraindicated product administered	535	23,520	7,978	388,472	5.1	0.024	1.11 (1.01–1.21)	0.147
TCZ + Methotrexate	Arthropathy	481	23,574	7,173	389,277	4.6	0.032	1.11 (1.01–1.22)	0.147
TCZ + Methotrexate	Synovitis	504	23,551	7,209	389,241	9.7	0.002	1.16 (1.05–1.27)	0.208
TCZ + Methotrexate	Treatment failure	629	23,426	6,952	389,498	123.7	<0.000	1.50 (1.38–1.63)	0.589
TCZ + Methotrexate	Therapeutic product effect decreased	401	23,654	5,487	390,963	5.8	0.016	1.21 (1.09–1.34)	0.273
**TCZ + Methotrexate**	**Stomatitis**	401	23,654	3,289	393,161	182.8	<**0.000**	2.03 (1.83–2.25)	**1.019**
TCZ + Leflunomide	Rheumatoid arthritis	907	17,799	11,964	362,477	153.8	<0.000	1.54 (1.44–1.65)	0.627
TCZ + Leflunomide	Drug intolerance	597	18,109	7,839	366,602	102.3	<0.000	1.54 (1.42–1.68)	0.625
TCZ + Leflunomide	Contraindicated product administered	442	18,264	7,301	367,140	15.7	<0.000	1.22 (1.10–1.34)	0.283
TCZ + Leflunomide	Arthropathy	375	18,331	6,749	367,692	4.1	0.043	1.11 (1.00–1.24)	0.156
TCZ + Leflunomide	Synovitis	231	18,475	6,343	368,098	22.8	<0.000	0.73 (0.64–0.83)	−0.463
TCZ + Leflunomide	Therapeutic product effect decreased	345	18,361	4,918	369,523	38.0	<0.000	1.41 (1.26–1.58)	0.498
TCZ + Hydroxychloroquine	Rheumatoid arthritis	984	18,390	11,902	355,470	193.3	<0.000	1.60 (1.49–1.71)	0.676
TCZ + Hydroxychloroquine	Drug intolerance	591	18,783	8,106	359,266	59.6	<0.000	1.39 (1.28–1.52)	0.480
TCZ + Hydroxychloroquine	Contraindicated product administered	487	18,887	7,166	360,206	30.1	<0.000	1.30 (1.18–1.42)	0.374
TCZ + Hydroxychloroquine	Synovitis	363	19,011	6,720	360,652	0.2	0.653	1.02 (0.92–1.14)	0.035
TCZ + Hydroxychloroquine	Therapeutic product effect decreased	351	19,023	4,755	362,617	37.8	<0.000	1.41 (1.26–1.57)	0.493
TCZ + Sulfasalazine	Rheumatoid arthritis	650	12,726	9,660	307,629	140.0	<0.000	1.63 (1.50–1.76)	0.702
**TCZ + Sulfasalazine**	**Drug intolerance**	597	12,779	6,922	310,367	300.7	<**0.000**	2.09 (1.92–2.28)	**1.067**
TCZ + Sulfasalazine	Contraindicated product administered	174	13,202	5,989	311,300	24.2	<0.000	0.69 (0.59–0.80)	−0.546
TCZ + Sulfasalazine	Arthropathy	332	13,044	5,771	311,518	31.2	<0.000	1.37 (1.23–1.54)	0.458
TCZ + Sulfasalazine	Synovitis	255	13,121	5,512	311,777	2.1	0.143	1.10 (0.97–1.25)	0.137
TCZ + Sulfasalazine	Therapeutic product effect decreased	274	13,102	4,005	313,284	62.1	<0.000	1.64 (1.45–1.85)	0.710
TCZ + Glucocorticoids									
TCZ + Prednisone	Rheumatoid arthritis	615	9,451	9,974	306,500	272.1	<0.000	2.00 (1.84–2.17)	1.000
TCZ + Prednisone	Joint swelling	370	9,696	7,437	309,037	73.5	<0.000	1.59 (1.43–1.76)	0.665
TCZ + Prednisone	Drug intolerance	308	9,758	5,825	310,649	78.7	<0.000	1.68 (1.50–1.89)	0.751
TCZ + Prednisone	Treatment failure	249	9,817	4,546	311,928	72.5	<0.000	1.74 (1.53–1.98)	0.799
TCZ + Prednisone	Maternal exposure druging pregnancy	180	9,886	3,851	312,623	26.1	<0.000	1.48 (1.27–1.72)	0.564
TCZ + Methylprednisolone	Rheumatoid arthritis	615	9,451	9,974	306,500	272.1	<0.000	2.00 (1.84–2.17)	1.000
TCZ + Methylprednisolone	Joint swelling	370	9,696	7,437	309,037	73.5	<0.000	1.59 (1.43–1.76)	0.665
TCZ + Methylprednisolone	Drug intolerance	308	9,758	5,825	310,649	78.7	<0.000	1.68 (1.50–1.89)	0.751
**TCZ + Methylprednisolone**	**Abdominal discomfort**	66	10,000	6,668	309,806	101.7	<**0.000**	0.31 (0.24–0.39)	**−1.705**
TCZ + Methylprednisolone	Hypersensitivity	185	9,881	5,322	311,152	1.4	0.231	1.09 (0.94–1.27)	0.130
TCZ + Methylprednisolone	Treatment failure	249	9,817	4,546	311,928	72.5	<0.000	1.74 (1.53–1.98)	0.799
TCZ + Methylprednisolone	Peripheral swelling	220	9,846	4,602	311,872	35.9	<0.000	1.51 (1.32–174)	0.599
TCZ + Methylprednisolone	Nasopharyngitis	186	9,880	3,692	312,782	38.6	<0.000	1.59 (1.37–1.85)	0.673
TCZ + Methylprednisolone	Musculoskeletal stiffness	180	9,886	3,851	312,623	26.1	<0.000	1.48 (1.27–1.72)	0.564
TCZ + Methylprednisolone	Hypertension	147	9,919	2,906	313,568	31.0	<0.000	1.60 (1.35–1.89)	0.677
TCZ + Methylprednisolone	Intentional product use issue	77	9,989	2,502	313,972	0.1	0.775	0.97 (0.77–1.21)	−0.048
TCZ + Dexamethasone	Condition aggravated	54	2,082	308	12,647	0.2	0.673	1.07 (0.79–1.43)	0.091
**TCZ + Dexamethasone**	**Infusion related reaction**	4	2,132	280	12,675	37.6	<**0.000**	0.08 (0.03–0.23)	**−3.558**
**TCZ + Dexamethasone**	**Infection**	23	2,113	300	12,655	13.4	<**0.000**	0.46 (0.30–0.70)	**−1.123**
TCZ + Dexamethasone	Intentional product use issue	28	2,108	175	12,780	0.0	0.882	0.97 (0.65–1.45)	−0.044
TCZ + NSAIDs									
TCZ + Celecoxib	Joint swelling	29	1,589	2,044	107,021	0.1	0.810	0.96 (0.66–1.38)	−0.066
TCZ + Celecoxib	Musculoskeletal stiffness	33	1,585	1,799	107,266	1.5	0.222	1.24 (0.88–1.76)	0.312
TCZ + Celecoxib	Mobility decreased	16	1,602	1,602	107,463	2.6	0.110	0.67 (0.41–1.10)	−0.578
**TCZ + Ibuprofen**	**Joint swelling**	51	966	3,216	130,249	28.9	<**0.000**	2.14 (1.61–2.84)	**1.096**
TCZ + Ibuprofen	Peripheral swelling	40	977	2,710	130,755	18.2	<0.000	1.98 (1.44–2.72)	0.982
TCZ + Ibuprofen	Infection	26	991	2,302	131,163	4.1	0.043	1.49 (1.01–2.21)	0.580
**TCZ + Naproxen**	**Joint swelling**	49	1,231	2,276	122,064	27.8	<**0.000**	2.13 (1.60–2.85)	**1.094**

AE, adverse event; CI, confidence interval; DDIs, drug–drug interactions; DMARDs, disease-modifying antirheumatic drugs; NSAIDs, non-steroidal anti-inflammatory drugs; PT, preferred term; ROR, reporting odds ratio; TCZ, tocilizumab.

Statistically significant results are highlighted in bold: log_2_ROR >1 or log_2_ROR <−1, and Pearson’s chi-square test with *p* < 0.05. ROR >1 indicates the male is more likely to have a higher risk of target AEs, and 0 < ROR < 1 indicates female tends to have a higher risk of target AEs.

For age groups, five drugs showed significant differences in target AEs related to DDIs, all of which were reported in patients aged ≥18 years. During TCZ in combination with specific drugs, patients aged ≥18 years were monitored as follows: infusion-related reactions with hydroxychloroquine, sulfasalazine, or methylprednisolone; synovitis and therapeutic product effect decreased with leflunomide; and joint swelling with naproxen.

For sex groups, six drugs exhibited significant sex differences in target AEs related to DDIs. Male patients demonstrated a higher incidence of stomatitis when TCZ was combined with methotrexate and of joint swelling when co-administered with ibuprofen or naproxen. In contrast, female patients showed a greater susceptibility to infusion-related reactions and infection when TCZ was combined with dexamethasone, and of abdominal discomfort when co-administered with methylprednisolone.

Moreover, no significant differences related to age or sex were observed for the AEs associated with DDIs of TCZ when used in combination with prednisone, celecoxib, or diclofenac.

## 4 Discussion

This is the first study to systematically evaluate the DDIs between TCZ and its most frequently co-prescribed medications in clinical practice. It provides a new reference for the safety monitoring of TCZ combined with 11 drugs in clinical practice, offering supportive information for clinical applications and contributing to the rationalization and safety improvement of medication use. The data for this study were obtained from the OpenVigil 2.1 platform, which provides streamlined access to the FAERS and offers significant advantages as a global spontaneous reporting database for pharmacovigilance research. These advantages include deriving real-world evidence from worldwide reports that enhances generalizability beyond clinical trial settings (14), providing an accessible analytical interface with preprocessed data that eliminates complex computational requirements (16), and possessing generation capacity for detecting rare adverse events and drug interactions often missed in premarketing studies. However, several limitations inherent to pharmacovigilance databases must be acknowledged, including potential underreporting, reporting biases, incomplete clinical information, and the inability to establish definitive causal relationships (17, 18). Therefore, results from OpenVigil should be considered as starting points for further research rather than as definitive proof of drug safety issues.

Although all four models belong to frequency-based signal detection methods, their underlying principles and statistical emphases differ. For example, the reporting ratio method is directly based on the number of reports, making it relatively intuitive but susceptible to bias. In contrast, the Ω shrinkage measure model reduces the influence of extreme values by introducing a shrinkage estimate, thereby improving stability in cases with small sample sizes. The combination risk ratio model integrates multidimensional risk ratio information and is theoretically more comprehensive, though it may be less stable when data are sparse. The chi-squared statistics model is based on the chi-squared test and is suitable for detecting signals with significant frequency differences; it shows a high degree of synergy with the Ω shrinkage model. Through pairwise consistency analysis (Table 3 and Figures 2a,b), we found that certain model combinations—such as the Ω shrinkage measure model and the chi-squared statistics model—exhibited strong agreement, suggesting a higher degree of synergy in signal identification. Therefore, in this study, we initially included all adverse events detected by at least one model for the analysis of drug interaction relevance. After obtaining the initial results, we focused specifically on those adverse events that were not only associated with drug interactions but also detected by at least two models.

Several AE records identified in this study, such as glossodynia, hand deformity, and rheumatic fever, were relatively rare. The results showed that all four frequency-based statistical models included in this study detected signals for the aforementioned rare AEs, and the corresponding RRR_diff values (relative reporting rate differences) for these AEs were significantly higher than the predefined threshold (RRR_diff >0), indicating a significant positive association with potential DDIs. Although these models are capable of identifying statistically significant associations from spontaneous reporting data, they cannot directly establish causality or elucidate definitive biological mechanisms. In addition, in spontaneous adverse drug reaction reporting systems such as the FAERS, the data originate from unstructured clinical practice reports. Consequently, the occurrence of extremely rare AEs (e.g., rheumatic fever) is more likely attributable to complex disease backgrounds, unrecognized concurrent infections, and ambiguous or erroneous diagnostic coding rather than a direct causal relationship with the drugs administered (19). Therefore, we advocate cautious interpretation of these AE signals and recommend that clinical practice place greater emphasis on common AEs that may have a causal relationship.

Glossodynia was primarily observed in reports involving the co-administration of tocilizumab (TCZ) and glucocorticoids, particularly prednisone. Although glossodynia is not a typical adverse reaction to TCZ or glucocorticoids, previous studies have documented glossodynia as an AE associated with tocilizumab (20). In addition, prolonged use of glucocorticoids may be associated with oral mucosal lesions or sensory disturbances, potentially through mechanisms involving local immunomodulation or neuroinflammatory processes (21). We suggest that, when patients report taste abnormalities,—especially during glucocorticoid therapy—clinicians should comprehensively evaluate their medication history and overall clinical status. Although current evidence remains limited, individual susceptibility or indirect effects of drug interactions cannot be excluded.

Hand deformity was predominantly identified among patients in the group receiving combination therapy of TCZ and disease-modifying antirheumatic drugs (DMARDs) (e.g., methotrexate, hydroxychloroquine, and leflunomide). Hand deformity is typically a late manifestation of rheumatoid arthritis (RA) resulting from disease progression. In our signal analysis, however, it was associated with an increased frequency of AE reports related to combination therapy. This association may reflect inadequate disease control, poor therapeutic response, or failure of the treatment regimen to effectively halt disease progression. It is unlikely to represent a direct drug-induced morphological alteration. Accordingly, we propose interpreting this as a potential monitoring signal for treatment response; when such reports emerge, clinicians should reassess the patient’s disease activity and the adequacy of the treatment regimen.

Rheumatic fever was associated with the concomitant use of TCZ and non-steroidal anti-inflammatory drugs (NSAIDs), particularly diclofenac. Rheumatic fever is an autoimmune inflammatory disorder triggered by infection with Group A β-hemolytic streptococcus (GAS), mediated through molecular mimicry-mediated immune cross-reactivity (22). Its appearance in AE reports is more plausibly attributed to complex underlying disease factors or misreporting. Nevertheless, its detection serves as a reminder for vigilance when NSAIDs are used for pain or inflammation control, as potentially unrecognized and uncontrolled infections (e.g., streptococcal) may precipitate immune-mediated systemic responses. We recommend that, for patients presenting with unexplained systemic inflammatory symptoms, potential infectious causes should be investigated and the indication for NSAID use carefully reassessed.

Notably, a strong positive correlation between anti-cyclic citrullinated peptide antibody (ACPA) positive and DDIs was observed when TCZ was used in combination with methotrexate, hydroxychloroquine, sulfasalazine, leflunomide, and diclofenac. Although anti-CCP is traditionally regarded as a diagnostic biomarker for rheumatoid arthritis (RA), it is categorized as a laboratory abnormality in the FAERS database and primarily classified under the “General Investigations” in SOC. When clinicians associate Anti-CCP positivity with drug therapy in reports (e.g., significant elevation of Anti-CCP titers), data abstractors map this to the corresponding MedDRA Preferred Term, categorizing it as an AE. This documentation reflects clinicians’ focus on treatment-related laboratory parameter changes rather than solely diagnostic utility. While Anti-CCP is a biomarker, its aberrant changes (e.g., treatment-emergent positivity or titer surges) may signal potential risks (e.g., treatment failure or disease progression), aligning with FAERS’ definition of an AE. To the best of our knowledge, there has been no relevant research for mechanism reported to date. ACPA is a specific marker for RA and is not only an indicator for the diagnosis but also a predictor of disease outcome (23). It was reported that higher levels of Anti-CCP antibody positivity were associated with more adverse clinical consequences (24). Therefore, patients with RA receiving the above combination therapy should be closely monitored for cutaneous and mucosal symptoms, with periodic reassessment of serological parameters, including ACPA.

When TCZ is combined with leflunomide in patients aged ≥18 years, attention should be paid to a potential decrease in therapeutic efficacy. A potential mechanism for the decreased treatment efficacy might be related to the formation of anti-TCZ antibodies; these antibodies have been reported as a common cause for lower drug serum concentrations and as poorer response to treatment (25). Previous studies have reported that 3–5% of patients were detected to have anti-TCZ antibodies (26). Moreover, in this study, co-administration of TCZ with hydroxychloroquine, leflunomide, sulfasalazine, or methotrexate showed the strongest DDI signals for increased C-reactive protein levels. The strongest DDI signals may be related to the production of anti-TCZ antibodies. Although treatment with TCZ dramatically reduced CRP concentrations in the plasma and CSF, the effect rapidly diminished in the weeks after treatment was stopped. Notably, a CRP reduction of <20% from baseline to 4 weeks of treatment on TCZ is a poor prognostic marker and should elicit considerations for changing treatment to another agent (28). In addition, fixed-dose might be a better dosing strategy for achieving a more consistent drug exposure compared to weight-based dosing (29).

Our study found that when TCZ is used in combination with ibuprofen or naproxen, joint swelling had the strongest correlation with drug interaction, and the incidence of joint swelling might be higher in males and adults. To the best of our knowledge, this result has not been reported in previous research. In contrast, TCZ inhibits the progression of joint damage by suppressing IL-6 signaling, while NSAIDs alleviate pain and inflammation in surrounding tissues through cyclooxygenase enzyme inhibition. This may be related to the TCZ binds to soluble IL-6R in a dose-dependent manner (30), and in patients with higher body weight or high disease activity, the standard dose (e.g., 8 mg/kg) may be insufficient to fully suppress the IL-6 signaling pathway, which may lead to the production of anti-TCZ antibodies in patients and result in higher disease activities. Therefore, it is essential that close monitoring be continued during dose reduction in the remission phase to ensure patient safety, while clinicians must remain vigilant for potential disease recurrence.

Sex is well known to influence risk, severity, and treatment outcomes of RA. Compared with male patients, female patients tend to exhibit higher disease activity scores, worse prognosis (24), and are less likely to achieve remission. Consistent with these findings, our study found that female patients showed a greater susceptibility to infusion-related reactions and infections when TCZ was combined with dexamethasone, as well as increased abdominal discomfort when co-administered with methylprednisolone. The most common AE associated with TCZ was infections, which may be due to TCZ being a direct inhibitor of the interleukin-6 receptor (IL-6), and inhibition of IL-6 was associated with immunosuppression (31). Nguyen et al. reported three comparable cases of patients with RA treated with TCZ who developed a severe prosthetic *Staphylococcus aureus* infection (32). Furthermore, C-reactive protein levels decrease rapidly after initiating TCZ treatment. During TCZ administration, both CRP and erythrocyte sedimentation rate (ESR) levels may remain low, potentially leading to infections that might not be recognized (33). It should be stressed that TCZ can suppress acute-phase reactions, such as fever and an increase in CRP, and induce transient decreases in neutrophil counts, thereby obscuring the signs and symptoms associated with infection, possibly leading to a delay in diagnosis (33). Therefore, the impact on CRP, neutrophils, and signs and symptoms of infections should be considered when evaluating a patient for a potential infection.

However, several noteworthy limitations inherent in this study should be acknowledged. First, the OpenVigil FDA platform lacks detailed patient-level information, especially regarding comorbidities and concomitant medication, which limits further assessment of patient disease status and AE severity. Second, the scope of entities eligible to submit AE reports is broad: not only healthcare professionals but also patients, their family members, patient representatives, and pharmaceutical manufacturers can submit AE reports. This may lead to duplicate or incomplete reporting. Third, in clinical practice, patients may concurrently use three or more suspect drugs. Finally, since some of the adverse events (AEs) included in this study lacked patient age and sex information in the database, and the database only allows filtering reports for a specific age range by setting minimum age and maximum age thresholds (in years), precise individual patient age could not be obtained. Thus, to some extent, it limited the subgroup analyses from comprehensively capturing all potential DDI-related AEs and may have introduced a certain degree of signal omission bias. However, the FAERS database can only extract AE information associated with the combination of two drugs, making it impossible to investigate interactions among three or more drugs. Despite the inherent limitations, analyses of real-world data, such as those collected in the FAERS database, may provide valuable reference information and complement findings from randomized controlled trials (34). Clinicians and pharmacists should remain vigilant for potential DDI-related AEs.

## 5 Conclusion

Healthcare providers should remain vigilant for potential DDI-related AEs when TCZ is used in combination with DMARDs, glucocorticoids, or NSAIDs. Particular attention should be given to signals of decreased treatment efficacy, which may be associated with the formation of anti-TCZ antibodies, as well as to musculoskeletal, cutaneous, and gastrointestinal events. Monitoring serological parameters (e.g., CRP and anti-CCP), skin/mucosal symptoms, and signs of infection is recommended during combination therapy to support medication safety.

## Data Availability

The original contributions presented in the study are included in the article/supplementary material, further inquiries can be directed to the corresponding authors.

## Footnotes

1
https://openvigil.sourceforge.net/
